# Updates in Hormone Replacement Therapy for Survivors of Gynecologic Cancers

**DOI:** 10.1007/s11864-025-01298-5

**Published:** 2025-03-05

**Authors:** Megan Gorman, Karin Shih

**Affiliations:** Division of Gynecologic Oncology, Department of Obstetrics and Gynecology, Zucker School of Medicine at Hofstra/Northwell, Northwell Health, New Hyde Park, NY 11040 USA

**Keywords:** Uterine cancer, Ovarian cancer, Cervical cancer, Hormone replacement therapy, Menopause

## Abstract

Symptoms of menopause and the sequelae of gynecologic cancer treatment can be severe in their physical and mental impact on patient quality of life. Survivors of certain gynecologic cancers – namely, early-stage, low-grade endometrial cancers; epithelial and germ cell ovarian cancers; and early-stage squamous cell cervical, vulvar, and vaginal cancers – as well as those who have undergone risk-reducing surgery for BRCA or Lynch syndrome mutations may safely use hormone replacement therapy (HRT). Treatment is ideally initiated in patients younger than age 60 or within ten years of menopause. The decision to start treatment should be made on an individualized basis after discussion of risks, benefits, and symptom severity with patients. Data suggest that the safest HRT regimens in this population include low-dose vaginal estrogen for the treatment of vulvovaginal symptoms, or low-dose systemic estrogen for the treatment of vasomotor symptoms, combined with progesterone in patients with an intact uterus. Therapies such as SSRIs/SNRIs, vaginal moisturizers, pelvic floor physical therapy, and psychosocial counseling should also be considered when appropriate for their effectiveness in managing menopausal symptoms without the potential risk of hormones.

## Introduction

### Menopause and Hormone Replacement Therapy

Menopause is defined as the cessation of menstruation after either the natural or surgical loss of ovarian function. Natural menopause is determined retrospectively after a woman has experienced amenorrhea for 12 months. The average age of menopause in the US is 51 years old, though the perimenopausal period and its associated symptoms begins years before the last menstrual period [1].

Pre- or perimenopausal patients undergoing treatment for gynecologic cancers will often undergo induced menopause secondary to cancer treatment. Induced menopause is the cessation of menstruation and ovarian function due to surgical removal of the ovaries, or damage to the ovaries from radiation or medical treatment. Approximately 40% of patients who are diagnosed with gynecologic malignancies are pre- or perimenopausal at the time of their diagnosis [2].

Symptoms of decreased estrogen and progesterone include vasomotor symptoms, mood changes, and vulvovaginal atrophy, though not all patients seek treatment. The symptoms of treatment-induced menopause may be more severe given their more sudden onset, and can have a more negative impact on quality of life.

Vasomotor symptoms, affecting approximately 80% of menopausal women, are characterized by episodes of flushing, redness, perspiration, and chills, often accompanied by anxiety or sleep disturbances. These symptoms are best treated with systemic estrogen alone (ET) or estrogen combined with progestin (EPT) [3]. While perhaps less effective, several studies have also now demonstrated a role for SSRIs and SNRIs in treatment of vasomotor symptoms [4].

Even among patients without a cancer history, systemic hormone therapy with ET and combined EPT carries certain risks, particularly for thromboembolism, cardiovascular disease, breast cancer, and endometrial cancer in the case of ET. These risks were highlighted after the release of the initial findings of the Women’s Health Initiative, leading to a significant decrease in the use of hormone therapy in the following decade [5, 6] and the recommendation to discontinue hormone therapy after five years. Currently, the American College of Obstetricians and Gynecologists (ACOG) and The North American Menopause Society (NAMS) recommend that the dose and duration of therapy be individualized [7].

Genitourinary syndrome of menopause (GSM), or vulvovaginal atrophy, affects up to 40% of menopausal women. Decreased estrogen levels result in vaginal dryness, itching, discharge, dyspareunia, thinning of vaginal tissue, bleeding, shortening and narrowing of the vagina, and increased urinary tract infections. Patients seeking treatment for GSM alone may be offered local vaginal therapy. rather than systemic therapy to minimize treatment risks. Despite variable rates of vaginal absorption and the theoretical risk of endometrial hyperplasia or cancer from unopposed estrogen, a 2008 Cochrane meta-analysis showed no association with local estrogen therapy and endometrial hyperplasia [8].

Progestin-only therapy, testosterone, or compounded bioidentical hormones are not recommended for the treatment of menopausal symptoms. While some data may suggest a benefit of progestin-only therapy in reducing vasomotor symptoms [9], given the limited data on its safety and concern for the potential risk of breast cancer, it is not recommended as a first-line therapy for management of vasomotor symptoms.

Testosterone is not currently FDA approved for use in women. Some data suggests that it may be beneficial for the treatment of sexual dysfunction in menopause [10, 11], however it should be noted that endogenous testosterone levels do not correlate with sexual function. Testosterone therapy has also not shown any benefit in the treatment of vasomotor symptoms. The use of testosterone also carries the risk of adverse effects including clitoromegaly and hirsutism as well as theoretical risk of malignancy secondary to androgen aromatization.

Bioidentical hormones are plant-derived chemicals that are chemically similar to naturally occurring hormones, frequently marketed to those patients who may have safety concerns about conventional prescription hormones. The use of compounded bioidentical hormones increased after the publication of the WHI data in 2002 raised questions about the cardiovascular risks of combined hormone replacement therapy. Given this lack of FDA regulation, compounded hormones have unknown purity, potency, and quality, and the absorption and bioavailability of these compounds is highly variable. ACOG and NAMS advise against the use of custom-compound bioidentical hormones when proven safe and effective treatments are readily available [12–15].

## The Women’s Health Initiative

The Women’s Health Initiative (WHI) is the largest women’s health study ever conducted and substantially impacted the understanding of hormone replacement therapy (HRT) in postmenopausal women, particularly with regard to its safety. Prior to the WHI, HRT was widely prescribed to manage menopausal symptoms and to prevent chronic conditions like osteoporosis and heart disease. Between 1993 and 1998, the WHI recruited over 27,000 women age 50–79 over 40 sites in the U.S. to assess the impact of HRT on coronary heart disease and breast cancer incidence; secondary outcomes also included endometrial cancer, colorectal cancer, stroke, PE, hip fracture, and death. The women received either conjugated equine estrogen (CEE) alone or estrogen plus medroxyprogesterone acetate (MPA) in those patients with a uterus in situ [16, 17].

The trial was stopped early due to an increased risk of cardiovascular events and breast cancer with CEE plus MPA. Subsequent long-term follow-up of 16,608 patients demonstrated that CEE plus MPA significantly reduced the risk of endometrial cancer compared to placebo [17].

Additional follow-up data presented in 2024 demonstrated that in women with prior hysterectomy, CEE alone significantly increased ovarian cancer incidence and mortality. In contrast, CEE plus MPA did not increase ovarian cancer risk in patients who did not have a hysterectomy [18]. As long-term analyses continue to inform our understanding of the risks of hormone replacement therapy in the general population, it is important to consider that this study was not originally designed to assess gynecologic cancer risk, and that hormone regimens are now available in lower doses than assigned to these patients. Furthermore, the average age of WHI participants was 63 years, which is significantly older than the average age of menopause in the U.S. and above the age at which initiation of HRT might typically be recommended in the cancer population. Thus, while the WHI has shed light on how HRT may increase the risk of developing gynecologic cancers in postmenopausal women, it does not inform management of menopausal symptoms in gynecologic cancer survivors.

## Hormone Replacement Therapy in Patients With Endometrial Cancer

The average age of diagnosis of endometrial cancer is 60, but approximately 25% of cases are diagnosed in premenopausal women. Treatment typically involves some combination of surgical management with hysterectomy and bilateral oophorectomy, chemotherapy, immunotherapy and radiation therapy. Hormone replacement therapy has historically been unavailable to women with endometrial cancer given the well-established relationship between unopposed estrogen and the development of endometrial cancer in the general population. However, research has shown that the risk to patients with early-stage disease may be minimal. A prospective study of over 1200 patients with stage I-II disease who received estrogen therapy vs placebo suggested no significant harm with ET after hysterectomy and bilateral oophorectomy. Though the study was incomplete and closed early as a result of the WHI, 2.3% of patients recruited who received systemic estrogen developed recurrence over a median follow up of 36 months, versus 1.9% of patients who received placebo [19]. A meta-analysis and a Cochrane review including this and several observational studies have drawn similar conclusions, suggesting that HRT is relatively safe in early-stage endometrial cancer survivors [20–22]. In contrast, the safety of HRT in patients with advanced endometrial cancer has not been studied or proven, and thus it is not recommended.

There is minimal research regarding the different outcomes of hormone therapy on different subtypes of endometrial cancer. Endometrial cancer has classically been divided into two groups: hormone-dependent types, such as endometrioid carcinoma, which account for the majority of cases, and non-hormone-dependent types, such as serous carcinoma, clear cell carcinoma, carcinosarcoma, and sarcoma. Despite this classification, many of these hormone-independent tumors do express estrogen and progestin receptors and have potentially beneficial response to antiestrogen therapy; thus, systemic hormone therapy is not recommended for patients with advanced hormone-independent cancers..

## Hormone Replacement Therapy in Patients With Ovarian Cancer

In the general population, a significant correlation has been demonstrated between exogenous postmenopausal estrogen use and increased ovarian cancer risk. Multiple large prospective cohort studies, including The Million Women Study [23], The Danish Sex Hormone Register Study (DaHoRS) [24, 25], and The Nurses' Health Study (NHS) [26] demonstrated an increased risk of ovarian cancer in women using HRT. Results of these and other studies have suggested that the risk might be greater with ET than EPT, and the risk may be more strongly associated with the development of endometrioid than serous or mucinous tumors. A more recent cohort study of 75,606 women in France, however, found no increased ovarian cancer risk with ET [27].

In the setting of these findings, providers have historically been hesitant to prescribe HRT to ovarian cancer survivors. However, multiple studies have demonstrated its safety in this population. A prospective study of 799 women in Sweden with epithelial ovarian cancer or borderline ovarian tumors showed that there was no difference in overall survival in patients with borderline tumors or with patients with epithelial cancers who used HRT before diagnosis. The data also suggested improved survival in epithelial ovarian cancer patients who used HRT after diagnosis [28]. A trial including 150 patients with epithelial ovarian cancer randomized to hormone therapy or control also showed an overall and relapse-free survival advantage with ET over a median follow up of 19 years [29]. A meta-analysis by Li et al. of two randomized control trials and four cohort studies also suggested that HRT has a favorable impact on overall survival in patients with epithelial ovarian cancer and was not associated with an increased risk of recurrence [30]. More recently, a prospective study of 1784 women in Korea also demonstrated a significant overall survival advantage to ovarian cancer patients receiving HRT [31]. Another review and meta-analysis of six studies concurred that among 1521 women, there was a significant reduction in ovarian-cancer related deaths among women who received HRT [32].

However, it seems that, as in endometrial cancer, different subtypes of ovarian cancer may have different risk profiles with HRT. Considering the known potential benefit of antiestrogen therapy in low grade serous and endometrioid ovarian cancers, HRT is not recommended for these patients. Patients with borderline tumors of the ovary may also be safely prescribed HRT, however there is limited research in this population, and is especially not suggested in those patients with high-risk features for evolution toward low grade serous carcinoma [29, 33]. In summary, while more high-quality randomized control trials are needed, estrogen therapy may not only be safe but beneficial in patients with high grade serous ovarian cancer.

## Hormone Replacement Therapy in Patients With Cervical Cancer

Squamous cell carcinoma of the cervix, the most common subtype of cervical cancer, is not a hormonally-driven cancer and ovarian conservation is recommended in patients who are premenopausal at time of diagnosis; as such, it is a reasonable to expect that HRT use might be safe in this population. In a prospective study of 120 patients with early-stage cervical cancer treated with surgery or radiation, there was no difference in five-year recurrence or overall survival in patients receiving either estrogen-alone or estrogen-progestin therapy vs placebo [34]. A 2021 systematic review suggested no increased risk of developing squamous cell carcinoma with hormone therapy, a weak increase in risk of adenocarcinoma, and improvement in quality of life, all favoring HRT in this population [35]. The safety of HRT in adenocarcinoma has also been questioned given the presence of estrogen receptors in these tumors. A recent small case–control study of 58 women with cervical adenocarcinoma did not show a statistically significant difference in those patients who received HRT compared to those who did not or who had ovarian conservation [36]. In summary, in those patients who do experience induced menopause after cervical cancer treatment, it is safe to use HRT for symptom management. Data are less clear for adenocarcinoma but suggest that HRT may be safe in those patients with early-stage disease [33]. Combination therapy is recommended for patients with a uterus in situ.

While there is a paucity of data to address the safety of HRT in vulvar and vaginal cancers, we can extrapolate from cervical cancer data given the similar tumor biology. Like cervical cancer, most vulvar and vaginal cancers are HPV-related squamous cell carcinomas and not hormonally driven. As such, it is reasonable to offer HRT to survivors of these cancers as well. There is no data to guide the use of HRT in rare vulvar or vaginal adenocarcinomas [37].

## Hormone Replacement Therapy in Patients After Risk-reducing Surgery

Patients with an increased genetic risk for gynecologic cancer due to BRCA mutation or Lynch syndrome typically undergo risk reducing surgery with bilateral salpingo-oophorectomy (rrBSO) and/or hysterectomy that induces menopause. Currently patients with BRCA1 mutations are recommended to undergo rrBSO at age 35–40, and those with BRCA2 mutations are recommended to undergo rrBSO at 40–45 [38]. Despite some conflicting data, most studies have shown that in patients without a personal history of breast cancer, a short course of estrogen therapy may help alleviate the symptoms of induced menopause without increasing the risk of breast cancer after oophorectomy. A prospective cohort study of 872 patients with BRCA mutation and no personal cancer history who underwent rrBSO had no increased risk of breast cancer overall with HRT, however the risk of breast cancer was greater with EPT vs. ET [39]. A more recent retrospective cohort of 306 BRCA mutation carriers who underwent rrBSO found a significantly elevated risk of breast cancer in patients over age 45 [40]. Patients with a uterus in situ should be recommended to use progesterone in addition to estrogen, weighing the relative risk of endometrial cancer and breast cancer. In patients who do have a personal history of hormone-sensitive breast cancer, hormonal therapy is not recommended due to risk of cancer recurrence [41–43]. Patients with Lynch syndrome – particularly those with MLH1, MSH2, MSH6 mutations – should be offered risk-reducing hysterectomy and BSO by their mid-40 s [44]. Patients with Lynch syndrome who have undergone prophylactic hysterectomy may use estrogen therapy alone for treatment of menopausal symptoms without increasing risk of colon cancer. There are no data to guide use of HRT in patients with Lynch syndrome who have not undergone hysterectomy. As genetic testing panels expand and identify new mutations that increase risk of hormonally-driven cancers, we may hope to collect more data about the risk of HRT in these populations as well.

## Recommended Treatment Regimens

Cancer survivors treated with hormone therapy should also follow the same general principles as those without a cancer history regarding treatment regimens: i.e., patients should be prescribed the lowest possible dose for the shortest period of time in order to achieve symptom management. Extrapolating from NAMS recommendations, treatment should be initiated by age 60 or within ten years of menopause in order to maximize the benefits of HRT, such as improved bone health, while minimizing the risk of adverse events like CAD, stroke, VTE, dementia, and breast cancer [45]. Particularly in the cancer population, HRT should be considered for those patients at or after the time of induced menopause to minimize symptoms and overall health risks of decreased estrogen. In those patients for whom HRT is appropriate, therapy should be tailored to the menopause symptoms experienced. Use of HRT should be especially considered in symptomatic patients who underwent early oophorectomy and may be at increased risk for health consequences of estrogen loss. Table 1 highlights selected options for hormonal therapy in gynecologic cancer survivors. Vasomotor symptoms of menopause can initially be treated with a standard dose, low dose, or ultra-low dose systemic estrogen; for example, oral low dose micronized estradiol-17B 0.5 mg/day or transdermal estradiol-17B 0.025 mg/day [7]. In patients with an intact uterus, progestin therapy (such as depot medroxyprogesterone acetate) should be given in addition to estrogen to minimize endometrial stimulation; there is currently insufficient data to definitively say that the levonorgestrel IUD is sufficient in this population. Symptoms should be reassessed after five years of treatment and continued based upon an assessment of individual clinical risks and benefits at that time. For patients primarily seeking relief of GSM, treatment can consist of vaginal estrogen alone, available in cream, tablet, or ring insert formulations. Vaginal estrogen restores an acidic pH in the vaginal microenvironment and improves blood flow, lubrication, and elasticity of the vaginal, reducing symptoms of dryness and dyspareunia [46]. Common regimens include the vaginal estradiol-17B 7.5 µg/day, estradiol vaginal tablet 10 µg/day, or conjugated estrogen cream 0.5–2 g/day. These regimens can be continued to maintain desired treatment effect. Patients seeking treatment for genitourinary rather than vasomotor symptoms should be treated with vaginal estrogen instead of systemic, as vaginal estrogen is associated with minimal systemic absorption and therefore may carry a decreased risk of adverse events [45]. Patients may also consider nonhormonal therapies like SSRIs and SNRIs, topical over-the-counter options like vaginal moisturizers, pelvic floor physical therapy, psychosocial therapy, and lifestyle modifications if menopausal symptoms persist (Table 2) [47, 48]. A recent survey of 61 Italian oncologists regarding attitudes toward prescribing HRT to gynecologic cancer survivors and BRCA mutation carriers revealed that while a majority of respondents reported discussing HRT with patients, there were differences in favor of prescribing HRT based on cancer history, with only 8.2% in favor of prescribing HRT for endometrial cancer [49]. This underscores the gap between theory and practice in HRT, emphasizing the need for clear guidelines to help physicians feel more confident in prescribing medications that can greatly improve the quality of life for gynecologic cancer survivors.

**Table 1:** Selected options for hormonal therapy in gynecologic cancer survivors for the management of menopause symptoms and treatment sequelae

Hormonal Management of Menopause Symptoms in Gyn Cancer Survivors	
Systemic Estrogen	
Standard Dose	Conjugated estrogen 0.625 mg/d Micronized estradiol-17β 1 mg/d Transdermal estradiol-17β 0.0375–0.05 mg/d
Low Dose	Conjugated estrogen 0.3–0.45 mg/d Micronized estradiol-17β 0.5 mg/d Transdermal estradiol-17β 0.025 mg/d
Ultra-Low Dose	Micronized estradiol-17β 0.25 mg/d Transdermal estradiol-17β 0.014 mg/d
Systemic Progestin (in combination with estrogen)	Depot medroxyprogesterone acetate, 400 mg Micronized progesterone 200 mg/d x12d/m
Vaginal Estrogen	Estradiol-17β ring 7.5 mcg/d Estradiol vaginal tablet 10 mcg/d Estradiol ring 0.05 mg/d Estradiol-17β cream 2 g/d Conjugated estrogen cream 0.5–2 g/d

**Table 2:** Selected options for nonhormonal therapy in gynecologic cancer survivors for the management of menopause symptoms and treatment sequelae

Nonhormonal Management of Menopause Symptoms in Gyn Cancer Survivors	
SSRIs/SNRIs	Paroxetine 7.5 mg/d Venlafaxine 37.5–75 mg/d Desvenlafaxine 100–150 mg/d Citalopram 10–20 mg/d
Alpha-2 Agonist	Clonidine 0.1 mg/d
GABA Analog	Gabapentin 600–900 mg/d
Vaginal lubricants Vaginal moisturizers	Silicone or water based lubricants Natural oils Polycarbophilic moisturizers Hyaluronic acid suppositories
Therapy and lifestyle changes	Cognitive behavioral therapy Pelvic floor physical therapy Acupuncture Exercise Avoidance of caffeine and alcohol

## Conclusion

The sequelae of gynecologic cancers and their treatments can include significant menopausal symptoms which can be challenging for patients. Clinicians must carefully assess the risks and benefits of hormone therapy in gynecologic cancer survivors and those with genetic increased risk of cancer. While further high-quality research is needed, there is sufficient evidence to suggest that HRT is safe in patients with early-stage endometrial cancer, epithelial ovarian cancer, and cervical cancer, according to the Society of Gynecologic Oncology (SGO) and ACOG [50–52]. While there has historically been hesitancy to prescribe hormone replacement therapy in cancer survivors and previvors, recent evidence suggests that HRT may be safe for some of these individuals. Any decision regarding HRT should be individualized, taking into account the patient's specific diagnosis, goals, and symptoms. Hormone therapy regimens used in cancer patients can be extrapolated from those prescribed to the non-cancer population, guided by the same principle of using the lowest dose for the shortest duration. Still, more robust clinical trials are needed to strengthen these recommendations and help establish clear guidelines for providers regarding optimal dose, duration, and route of treatment. Further research is crucial to understand how HRT risks differ among various cancer subtypes, as well as how other factors—such as patient BMI, newly identified genetic mutations, and ideal hormone doses specific to the cancer population—might modify these risks. For patients for whom HRT presents an unacceptable risk, alternative treatments should be considered, including SSRIs/SNRIs, topical therapies, and lifestyle modifications; but in general, HRT appears to be safe and underutilized for patients with early-stage endometrial cancer, high-grade serous ovarian cancer, and cervical cancer.

## Key References

- Chlebowski RT, Aragaki AK, Pan K, et al. Menopausal hormone therapy and ovarian and endometrial cancers: Long-term follow-up of the Women's Health Initiative randomized trials. J Clin Oncol. 2024;42(30):3537–49. 10.1200/JCO.23.01918.
  - ○ Analysis of long-term follow-up data from WHI assessing risk of ovarian cancer and endometrial cancer after hormone therapy.
- Fournier A, Cairat M, Severi G, Gunter MJ, Rinaldi S, Dossus L. Use of menopausal hormone therapy and ovarian cancer risk in a French cohort study. J Natl Cancer Inst. 2023;115(6):671–9. 10.1093/jnci/djad035.
  - ○ A recent cohort study that found no increased ovarian cancer risk with estrogen therapy.
- Villa P, Bounous VE, Amar ID, Bernardini F, Giorgi M, Attianese D, Ferrero A, D'Oria M, Scambia G. Hormone replacement therapy in post-menopause hormone-dependent gynecological cancer patients: A narrative review. J Clin Med. 2024;13(5):1443. 10.3390/jcm13051443.
  - ○ A recent review of HRT in patients with hormone-driven gynecologic cancers.
- Vargiu V, Amar ID, Rosati A, Dinoi G, Turco LC, Capozzi VA, Scambia G, Villa P. Hormone replacement therapy and cervical cancer: A systematic review of the literature. Climacteric. 2021;24(2):120–7. 10.1080/13697137.2020.1826426.
  - ○ A recent review of HRT in patients with cervical cancers.
- “The 2022 Hormone Therapy Position Statement of The North American Menopause Society” Advisory Panel. The 2022 hormone therapy position statement of The North American Menopause Society. Menopause. 2022 Jul 1;29(7):767–794. 10.1097/GME.0000000000002028.
  - ○ A recent discussion of screening and treatment options for sexual dysfunction in women affected by cancer.
- Sinno AK, Pinkerton J, Febbraro T, et al. Hormone therapy (HT) in women with gynecologic cancers and in women at high risk for developing a gynecologic cancer: A Society of Gynecologic Oncology (SGO) clinical practice statement: This practice statement has been endorsed by The North American Menopause Society. Gynecol Oncol. 2020;157(2):303–6. 10.1016/j.ygyno.2020.01.035.
  - ○ Practice statement outlining SGO recommendations for the use of HRT in patients with gynecologic cancers.
- Robison K, Kulkarni A, Dizon DS. Sexual Health in Women Affected by Gynecologic or Breast Cancer. Obstet Gynecol. 2024;143(4):499–514. 10.1097/AOG.0000000000005506.
  - ○ A recent discussion of screening and treatment options for sexual dysfunction in women affected by cancer.

## Data Availability

No datasets were generated or analysed during the current study.
